# A New Anæsthetic

**Published:** 1883-04

**Authors:** 


### "A NEW ANÆSTHETIC."

M. Bert discovered that prolonged anæsthesia could be induced by a mixture of nitrogen protoxide, 85 volumes, with oxygen, 15 volumes, if the application were made in a closed chamber and under certain pressure. M. de St. Martin, says the *Journal of Chemistry*, has obtained the same effects at ordinary pressure, by adding to the mixture a small quantity of chloroform. M. de St. Martin has not made a very astounding discovery, as a certain class of dentists have, under the name of "Vitalized Air," for some time employed a mixture of chloroform vapor with nitrous oxide, to produce anæsthesia. With this a state of narcotism is rapidly induced, and it may be maintained for some time—if enough of chloroform or ether is used. But so far from its providing any additional immunity from danger, it adds to any possible hazards from the nitrous oxide gas, all the risks attendant upon the other agent. That the former will axphyxiate, every intelligent dentist knows; and hence in giving it he pushes the administration to the utmost limit that anæsthesia may be reached before suffocation ensues. Any medical man can estimate the relative safety of such a method of giving chloroform or ether, even though the vapor may be diluted with another gas.

